# Communication when it is needed most—the past, present and future of volcano geoheritage

**DOI:** 10.1007/s00445-022-01574-4

**Published:** 2022-06-17

**Authors:** John Stix, Grant Heiken

**Affiliations:** 1 Department of Earth and Planetary Sciences, McGill University, 3450 University Street, Montreal, Quebec, H3A 0E8 Canada; 2 Freeland, WA 98249 USA

**Keywords:** Volcano geoheritage, Knowledge webs, Learning tools, Research integration

## Abstract

Our understanding of volcanoes and volcanic systems has been communicated through legends maintained by indigenous communities and books and journal articles for the scientific community and for the public. Today we have additional means to communicate knowledge and information, such as social media, films, videos and websites. To build on these mechanisms, we propose a comprehensive system of information collection and dissemination which will impact and benefit scientists, officials and politicians, students and the public at large. This system comprises (1) an information web for broad understanding of volcano systems and volcanology, and (2) a second web for individual volcanoes. This integrated geoheritage approach provides a template for information dissemination and exchange in the twenty-first century.

## Introduction

Volcano geoheritage generally refers to a geographical site that is generally accepted to have significant volcanological value in terms of intrinsic and/or cultural significance (Németh et al. 2017). In this contribution, we use the term volcano geoheritage in a different fashion which encompasses knowledge bases, data sources and other information related to volcanoes and volcanology. We examine how this knowledge is transmitted and communicated to the broader scientific and public communities, including past practices, current trends and future directions.

Over the last two centuries, advances in volcanology have resulted from tireless observations and communications with colleagues via professional meetings, personal discussions and teaching. The actual *heritage* is left in the form of the subsequent communications, be they lectures, papers, books or blogs. These materials are digested and passed on via cited materials to today's research.

Life has changed. Earlier influential volcanologists spent more time on their research and the resulting product was usually a book, for example Jaggar (1947), rather than the frequent journal articles that we now publish every year. We sometimes view our contemporary "heritage" as a means to justify the next research proposal. We should seriously consider taking more time to think about our research and putting the results into a significant volume or website for lasting impact. Those of us who publish extensively might examine who actually reads and cites our work. Will your research results survive into the future?

### The past 200 years

Volcano geoheritage was generated by a small but influential international group of volcanologists whose contributions fell into the following categories:

1. *Textbooks*

We still find pioneering ideas published in English such as Rittman (1962), MacDonald (1972) and Williams and McBirney (1979). Classic studies were also published in French (La Croix 1904), Italian (Mercalli 1907) and German (Zollinger 1855; Sapper 1927). Continuing in this tradition are the texts by Blong (1984), Cas and Wright (1987) and Francis (1993).

2 *General interest books*

These are books that were written to educate the public and students. This category includes works by Jaggar (1945), Decker and Decker (1981), Rosi et al. (2003), Oppenheimer (2011) and Fisher et al. (1997). Some of the most influential books for the public were the beautiful and dramatic "coffee-table books" by Maurice and Katia Krafft, who also produced an influential video on volcanic hazards (Krafft et al. 1995). Volcano encyclopedias include the *Encyclopedia of Volcanoes,* edited by H. Sigurdsson et al. (2000, 2015), and *Volcanoes and the Environment* (Martí and Ernst 2005).

3 *Specialty books*

These include books on igneous petrology (Carmichael et al. 1974; Hatch et al. 1973), and pyroclastic rocks (Fisher and Schmincke 1984; Heiken and Wohletz 1985; Westgate and Gold 1974). An accessible book on geothermal energy by Wohletz and Heiken (1992) is still available online for free. A particularly comprehensive book on the geophysical monitoring of volcanoes was published by Dzurisin (2007) and a guide to thermal remote sensing of volcanoes by Harris (2013). A comprehensive guide to volcanic hazards by Blong (1984) covers physical volcanology, social impacts, and effects on economic activity, with the main goal to reduce losses, both human and infrastructure. *Volcanic Plumes* by Sparks et al. (1997) covers theory, experimentation and observation of volcanic eruption plumes. The development of ideas regarding volcanic eruptions is well presented by Sigurdsson (1999).

4 *Historical volcanic eruptions that have influenced our scientific understanding*

Progress in understanding volcanoes has often followed specific volcanic eruptions, many with world-wide effects and/or great loss of life.

#### Vesuvius, Italy CE 79

The eruption was described by Pliny the Younger in letters to colleagues that have since been compiled and published (The Younger Pliny, translated by Radice 1963). These writings include the origin of the term "Plinian eruption."

#### Vulcano, Italy 1880

Mercalli and Silvestri (1891) published a very detailed, complete report on the Vulcano eruption of 1880.

#### Krakatau, Indonesia 1883

Comprehensive descriptions by Verbeek were published in 1885 and sponsored by the Dutch Government. A more familiar book to most of us, published in English in 1888, is the British Royal Society Report on the eruption of Krakatoa (English spelling). On the 100th anniversary of the Krakatau eruption, Simkin and Fiske (1983) included both reports, plus every journal article that had been published until 1983. The Krakatau eruption has provided basic understandings of caldera formation, volcanic tsunamis, volcano sounds, pumice fall and drift and global atmospheric effects. It has also been the basis for some fictitious movie fantasies, including "Krakatoa, East of Java."

#### Laki Fissure, Iceland, 1783-1784

Global circulation of ash and gases created a blue haze over Europe, which led to an unseasonably cold summer and subsequent famine. The connection between these atmospheric effects and the Laki eruption was made by Benjamin Franklin when he was the US ambassador to France. In Iceland, the eruption was described by Steingrimsson (translated in Steingrimsson 1998). Modern publications that describe the eruption have been published by Sigurdsson (1982) and Rampino and Self (1984).

#### Mont Pelée, Martinique, 1902

This eruption was described initially by the French volcanologist Alfred La Croix (1904). LaCroix was one of the first to describe pyroclastic density currents (nuées ardentes) and their catastrophic effects on the town of St. Pierre. Mont Pelée was also where rapid dome growth was observed in 1902 and again in 1929-1932 (Perret 1937).

#### Parícutin, Mexico, 1942-1952

This was an event where the birth of the volcano from a fissure in a cornfield led to a long-lived eruption that produced a large scoria cone and lava flows that engulfed the villages of Parícutin and San Juan Parangaricutiro. Publications by Wilcox (1954) Fries Jr (1953) and Fries Jr and Gutiérrez (1950) documented the eruption. Later studies focused on the effects of the eruption upon human, animal and plant communities. These publications and later studies were assembled into one volume by Luhr and Simkin (1993) in honor of the eruption's 50th anniversary.

#### Kilauea and Mauna Loa, Hawaii, USA

Frequent eruptions of these Hawaiian volcanoes have provided us with a steady flow of influential publications since the nineteenth century, beginning with Dutton (1884), followed by Dana (1890), Brigham (1909), followed by Jaggar and the staff of the Hawaiian Volcano Observatory (HVO). Today, HVO’s communications activities are mostly online but also include a remarkable record of publications, for example, descriptions of recent activity along Kilauea’s east rift. An understanding of these volcanoes can be found in the massive volumes *Volcanism in Hawaii* (Decker et al. 1987). We owe what we understand about most aspects of active basaltic volcanism to publications about Hawaii. Beginning with Jaggar, most geophysical observations of eruption precursors and subsequent eruptions were developed at the HVO (Apple 1987).

#### Mount Saint Helens, Washington state, USA, 1980-2006

The 1980 eruption of Mount St. Helens captured headlines for a long time and led to a new standard for interdisciplinary volcano studies that continues today. New geophysical techniques for volcano deformation and forecasting were established on the fly as the eruption continued. Much of what was learned was later published in a tome edited by Lipman and Mullineaux (1981). Continued activity (mostly dome growth) from 2004 to 2006 was described in an equally weighty volume edited by Sherrod et al. (2008). The Mount St. Helens eruption changed world views on sector collapse, blast effects and volcanic mudflows (lahars) and hazard mitigation.

#### Mount Pinatubo, Philippines, 1991

The explosive eruption of Mount Pinatubo, the second most voluminous eruption of the twentieth century, threatened a million people in the surrounding Philippine countryside and was forecast during the rapid response of volcanologists from the Philippines and the USA (Newhall and Punongbayan 1996). A quick study of previous eruptions provided an idea of the extent of the deposits from earlier eruptions. The residents were a bit skeptical about the forecasts, but a showing of a volcanic hazards video (Krafft et al. 1995) initiated a mass evacuation. Pinatubo's eruption left thick pumice and ash deposits, which were quickly eroded during heavy rainfall from a typhoon, generating fast-flowing lahars. The countryside was devastated and commerce affected (Rodolfo 1995). This was an eruption that resulted in interdisciplinary studies of the effects of volcanic eruptions on the population, an approach that has since been used at many eruptions around the world. Much of what was learned about the Pinatubo eruption was published in an 1100-page book edited by Newhall and Punongbayan (1996).

#### Galunggung, Indonesia, 1982

On June 24th, 1982, a British Airways flight from Kuala Lumpur, Malaysia, to Perth, Australia, flew into a volcanic ash plume from Galunggung volcano, Java. The aircraft's windows were sandblasted and all four engines shut down. Thanks to excellent piloting, the engines were restarted to make an emergency landing in Djakarta (Casadevall 1994). This event was a wakeup call to the aviation industry and to volcano observatories about the dangers of volcanic eruption plumes.

In 1986, the International Civil Aviation Organization created a volcanic ash warnings (VAW) study group. The VAW study group met in Montreal, Canada, to establish standards and rules for flights near volcanoes. The VAW study group built the framework needed to bring together volcano observatories, meteorological observatories, flight controllers, airlines and pilot associations. One of the VAW’s goals was to establish an interdisciplinary meeting of volcanologists, aircraft makers, pilots and flight control experts, which was held in 1991 (Casadevall 1994). Using the flight rules established by the VAW, plus more sophisticated ways of observing eruption plumes, later prevented potential accidents.

5 *Effects of eruptions on life, society and infrastructure*

An understanding of volcanic deposits has been important to archeologists for two reasons: 1. preservation of remains and artifacts and 2. the use of volcanic ash beds to date fossils, a common practice used by paleoanthropologists. For example, working in the Ethiopian rift, where fossil remains date back to 6 million years, understanding the tephrochronology was crucial to understanding the ages and paleoenvironment of these early hominins (Woldegabriel et al. 2000). Early works about volcanoes and archeology in Central America were published by Sheets (1983) and at sites across the world (Sheets and Grayson 1979).

Cultural legends about volcanoes were summarized in "Legends of the Earth" by Vitaliano (1973). Myths in many cultural groups in Papua New Guinea about "the time of darkness" were linked to a widespread eruption cloud from Long Island Volcano about 300 years ago (Blong 1982).

Myths abound about the effects of the Lower Bronze Age eruption of Thira (Santorini) during the seventeenth century BCE and include stories of Jason and the Argonauts and the origin of "Atlantis." This is a story that involves volcanology, archeology, mythology and the possible end of Minoan culture (Luce 1969).

Volcano geoheritage has become an important means to understand a wide variety of historical and mythological events. Major changes in everyday human activities have followed many volcanic eruptions, a topic covered comprehensively by Blong (1984).

6 *Adventure and volcano tourism*

When one of us (GH) was a boy, he was intrigued and stimulated by Haroun Tazieff's "Craters of Fire" (1952). In hindsight, it is evident that Tazieff was an active volcanologist but attracted the public's interest mostly because he was a "daredevil scientist." Most of us now approach volcanoes with caution; this is excellent for self-preservation but somewhat boring for the public. A great contemporary account of volcanologists and the problems faced while studying eruptions is by Dick Thompson, a writer for *Time* magazine, who published "Volcano Cowboys" (Thompson 2000). It is a superb contribution to volcano geoheritage by a journalist.

Many of the eruptions of Vesuvius in the 1700's (CE) were observed by Italian scientists but became most famous across Europe because of publications by Sir William Hamilton, the British Envoy to Napoli (1768-1795). Napoli and Vesuvius became an important destination for well-educated (and wealthy) Europeans as part of the "Grand Tour" during the late 1700’s and 1800’s. A modern history of Vesuvius, including the "Grand Tour," was published by Scarth (2009). Visitors included J. W. Goethe, writer and philosopher, who wrote about the eruptions of Vesuvius in *Italian Journey* (Goethe 1816-1817). A modern heritage from the Grand Tour has been the thousands of contemporary tours to Vesuvius, Etna and Stromboli, although these are mostly organized by travel agents and travel bureaus.

A tour guide to volcanoes in America's National Parks was published by Decker and Decker (2001)

7 *Volcano histories and eruption reports*

When the International Association of Volcanology was formed in 1922 (IAV, now IAVCEI) (Cas 2022), one of its goals was to catalog active volcanoes and their eruptions. The product was a series of monographs by region, which were published from 1951 to 1973; the volumes were valuable but incomplete, and many countries were left out. The limited number of these monographs have reduced their heritage value. Early reports of eruptions in Hawai'i were sent out as the Volcano Letter; all of the Volcano Letters were later organized into one volume by Fiske et al. (1987). The Smithsonian Institution sent out notices about recent eruptions from the Center for Short-Lived Phenomena. A more lasting heritage came in the form of the *Volcanoes of the World* by Simkin et al. (1981), which was updated in 1994 (Simkin and Siebert 1994) and again more recently (Siebert et al. 2011).

An important and useful heritage are the many volcano hazard maps published by geological and volcanological surveys and universities. Many are based on maps of previous deposits and some based on modeling of eruption phenomena. The hazard maps that are most effective are those that can be understood by the public in areas at risk. We explore these in more detail below.

## The present and the future

The *heritage* we are obligated to leave is the foundation for the next generation of volcanologists. What parts of this heritage will survive? Will these include lectures and training, websites, Facebook pages, Zoom meetings, journal articles, books, or something else? What is the “preservation potential” of such materials? In some ways this is challenging since there are now hundreds of volcanologists who each have their own means of communication. There is a myriad of websites, blogs, both professional and commercial journals, and yet there is still a substantial publication base (both hard-cover and e-books).

We also have the challenge of communicating to the public factual, interesting and exciting materials. Most everyone, especially children, are stimulated by material about volcanic eruptions. How do we communicate these events without "dumbing them down"?

The various lines of communication described above have served us well in volcanology for better understanding volcanoes and communicating this understanding. Today we are in an enviable position of having access to many new and exciting approaches to communicating knowledge at many different levels, including online learning which has been accelerated by the COVID-19 pandemic. People are actively exploring novel means and ways to transfer knowledge and understanding (Peltier et al. 2021). In designing and developing these new approaches, we should be mindful of several key principles and objectives that should guide us in our efforts. First, access to information should be simple and easy for all. Carefully designed media, e.g., websites, books, datasets and films to illustrate several examples, are important means of communication. They need to be designed so that they are widely used. Second, the principles of Equity, Diversity and Inclusion (EDI Principles) should form a fundamental pillar when designing new material. It is challenging yet essential that everybody has equal access to materials and information. This point is discussed in detail below. Third, in the design and implementation of new communication approaches, we must keep in mind that much of the world has limited resources. Given this reality, how can we best provide access to information for people and institutions with limited resources?

The 2020–2022 COVID-19 Pandemic provides an excellent template and opportunity to examine these issues. The pandemic has severely restricted our activities in every way imaginable and unimaginable. We have not been able to travel. We have not been able to physically meet and work with colleagues. Data collection has been difficult and, in some cases, impossible. Practically everything has been slowed down and made more difficult. Yet these limitations have created alternative mechanisms which work effectively, most notably virtual meetings at all scales. In this sense, the world has become a more equal and equalized environment, and we should capitalize on these developments as we push forward into the future.

With the Pandemic, therefore, we are at a fundamental crossroad in time which presents us with an opportunity to take new directions and try new approaches for communicating volcano geoheritage. Many of the ideas that we propose in the following pages are not new; a number of researchers have thought deeply about this subject. Our goal here is to provide a holistic and integrated view of communicating this geoheritage during the twenty-first century.

There are a number of interesting starting points. For example, Professor Bill Rose of Michigan Technological University has envisaged a comprehensive and central source of information for volcanoes, one where somebody can quickly and easily access and obtain key data and knowledge on a volcano (see for example https://pages.mtu.edu/~raman/VFuego/VFuego/Welcome.html for an excellent compendium on Fuego volcano in Guatemala). Another important central source of information is Vhub (https://vhub.org/). Here we propose to develop and expand this concept in a number of ways, leading to a comprehensive and linked information web for volcanoes and volcanology.

For this information web, we propose two approaches which are linked at different scales. The first web comprises a general compendium on volcanoes, volcanism and volcanology, including books, films and educational resources, to cite but three of many components which are explained in more detail below.

The second web is constructed for individual volcanoes, prioritizing those which pose the highest hazard and risk, e.g., the Decade Volcano program of the 1990’s (IAVCEI Subcommission on Decade Volcanoes 1994). This second web includes hazard, risk and vulnerability maps, digital elevation models (DEM’s) and educational videos, again to list only several of many elements which are described below.

By taking this approach, we would be in an excellent position to decide upon the truly important modes of communication, in the broadest sense, when the next large eruption occurs. These enhanced modes of communication will benefit our understanding of such eruptions, our preparation and our mitigation of these events. We might consider planning for two timescales of eruptions, a basaltic type which occurs on a decadal basis (e.g., Miyakejima 2000, Bárðarbunga 2014-2015, Kilauea 2018), and another larger, less frequent and more silicic type of eruption (e.g., Katmai 1912, Pinatubo 1991). Both the unrest associated with such eruptions and the scientific advances to be made in the future provide opportunities for us now to design the most effective means to disseminate knowledge, data and information flowing from these dynamic systems. The details for each web are discussed in the following sections.

## An integrated web of knowledge and communication for volcanoes and volcanology

Placing a date on the birth of modern volcanology is difficult, but we can safely say that volcanoes have been studied intensively for more than a century, with accelerations after significant eruptions. Careful quantification of eruptions and eruptive products perhaps began with the pioneering work of George Walker in the 1960’s and 1970’s. However, we must at the same time also acknowledge that many older and prehistoric societies worldwide have both lived with volcanoes and also conducted direct observation-based "research" on volcanoes (Vitaliano 1973).

Today we have an outstanding knowledge base, comprising observational, experimental and theoretical aspects, which provides a means to evaluate, study and understand volcanism in its many manifestations. These include processes taking place in the subsurface (e.g., magma chambers, intrusions, conduits), at the surface (e.g., pyroclastic flows, lava flows, lahars), and in the atmosphere and oceans (e.g., tephra dispersal, volcanic gases, ice cores). This knowledge base therefore serves as the central reference point into which a series of component parts feed different elements, which together contribute to a holistic vision of volcanoes and volcanology for the future.

**Fig. 1:**
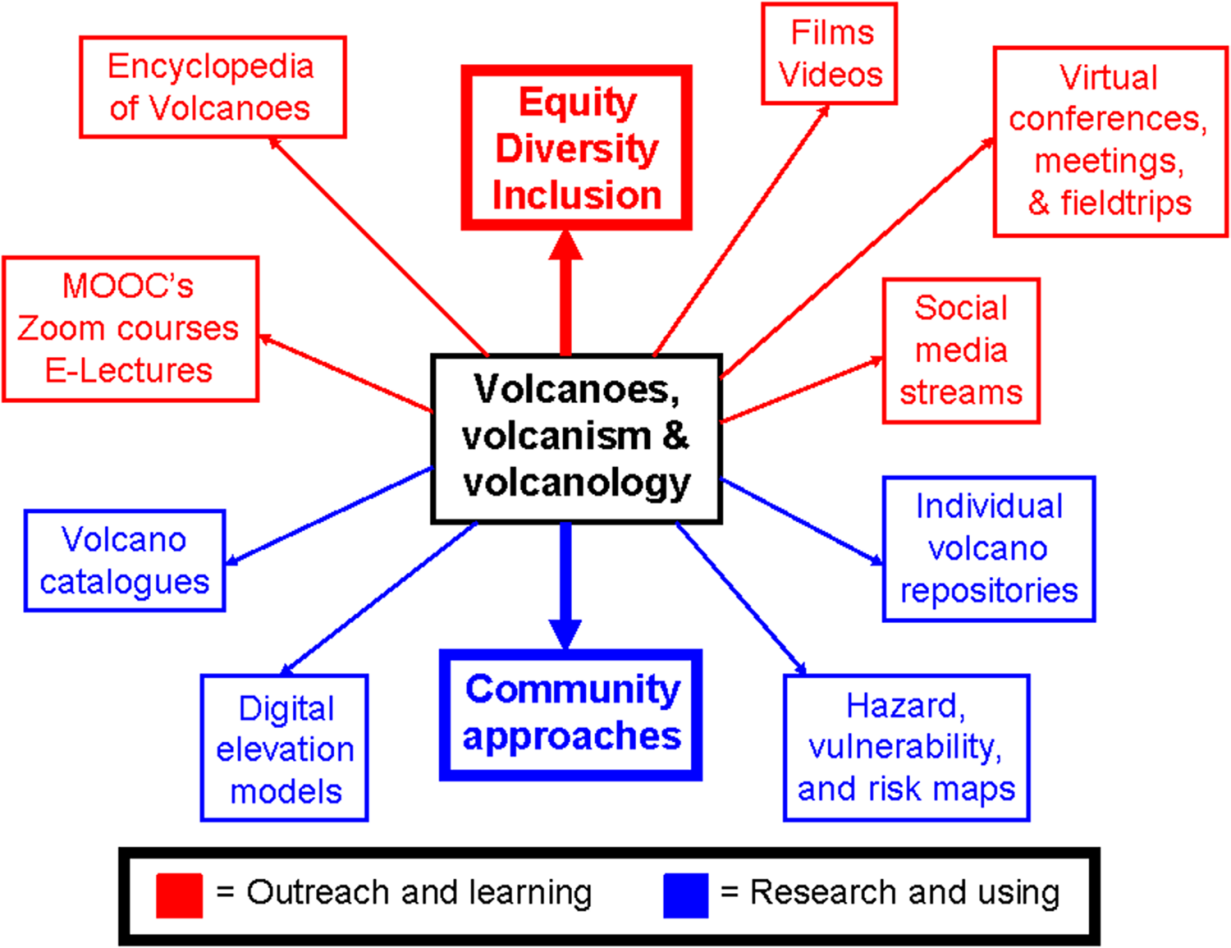
An integrated knowledge web for volcanoes, volcanism and volcanology

The component parts are twofold in nature. The first group involves "outreach and learning" and includes a means by which individuals and groups can gain knowledge about volcanoes, volcanism and volcanology. Many of these components are interactive in nature. The second group involves "research and using" components which comprise a series of information and data sets which we consider essential to the study of volcanoes. We now examine these individual "outreach and learning" and "research and using" components, making reference to Fig. 1, which displays our concepts in schematic and graphical form.

### Outreach and learning

#### Equity, diversity and inclusion (EDI)

When we communicate and access information related to volcanoes, volcanism and volcanology, the concepts of equity, diversity and inclusion are important to consider. In terms of public access to communication and information, it is essential to identify and prioritize marginalized groups that are both underrepresented in community decision-making processes and disproportionally impacted by disasters. These are the people who suffer the most and lose the most, whether they are living in the global north or the global south, and include women and girls, the very young and the very old, the disabled and injured, the socioeconomically disadvantaged, political and economic refugees, as well as indigenous, racial and ethnic minorities. Access to communication and information is not a one-way street. While under-represented individuals and communities need to be prioritized in terms of access to information, they can offer significant knowledge and insight into eruptions and unrest. For scientists, the same EDI principles discussed above are relevant. Key issues include traditional vs. non-traditional roles of scientists, gender parity at all levels, citizen science and leadership roles, including but not limited to decision-making, editorships of books and journals and heading organizations. The topic of EDI is a difficult one at times, yet one which needs to be a core principle for communication and volcano geoheritage.

#### The encyclopedia of volcanoes, 3rd edition

The current second edition of the *Encyclopedia of Volcanoes,* published in 2015, is the principal reference resource for practicing volcanologists, graduate students and others (Sigurdsson et al. 2015). This is because the Encyclopedia is both comprehensive and detailed in its coverage of volcanism, making it a key reference work for the volcanological community today. The Encyclopedia includes 78 chapters on a full range of topics related to volcanology. Hence this 1456-page compendium is an ideal starting point for exploring a topic related to volcanoes and volcanism. What might a third edition look like? It would likely remain comprehensive. Ideally it would be fully open access, allowing everyone equal access to the information contained within. It would be easily accessible electronically. It could also be constructed to be easily updated, i.e., a volcano wikipedia with a moderator or moderators as appropriate. Finally, it would be fully integrated into the "outreach and learning" component that we are proposing here. Each of the 78 chapters could be linked to (a) a learning module and (b) a video module, which are described below. This type of integration could redefine the concept and meaning of an encyclopedia in the twenty-first century.

#### Massive open online courses (MOOC’s), zoom courses and E-lectures

Since the Encyclopedia of Volcanoes covers 78 topics, we propose a series of MOOC’s, Zoom courses and e-lectures which cover all these topics. Although practically this proposal is challenging, in principle it is straightforward. Creating such content could be highly flexible. For example, one topic might be covered by a 10–20 minute lecture, another might require an hour's lecture, while others could be examined in greater detail by a package of lectures or courses. A particular topic could be treated in a number of ways, e.g., one course aimed for the public and schools, another at the university and college level, and yet another for professionals and practitioners. As with the Encyclopedia above, these courses should be available to all for free or a small fee, they should be accessible (e.g., closed captioning), and they could be multilingual for greatest impact globally. Such efforts have already begun; examples include a MOOC on magma movement (https://www.edx.org/course/monitoring-volcanoes-and-magma-movements), another on physical volcanology (http://www.ipgp.fr/en/physical-volcanology-mooc), a third on material properties (https://www.my-mooc.com/en/mooc/volcano/), and a fourth on volcanoes in Iceland and New Zealand (https://www.edx.org/course/exploring-volcanoes-and-their-hazards-iceland-and-new-zealand), to cite several interesting examples. The Natural Disasters MOOC at McGill (https://www.edx.org/course/natural-disasters) was conceived and assembled for active learning, with alternating components of lectures, activities, quizzes, demonstrations and interviews on a particular topic (Stix et al. 2018).

#### Films and videos

Following the logic outlined above, films and videos could be produced for each of the 78 topics, again with the same flexibility in mind, ranging from short 3–10 minute video clips to hour-long in-depth examinations of phenomena. As with courses and MOOCs, videos and films could be produced for different audiences. Open access, accessibility and multilingual principles would be over-arching goals for maximum impact. An excellent example are the recent series of short videos on Vimeo covering topics such as pyroclastic flows, lahars and gases, which are produced in a number of languages and freely available to all (https://vimeo.com/volfilm).

#### Virtual conferences, meetings, workshops and fieldtrips

New and provocative ideas are proposed, discussed and examined at on-site conferences, meetings, workshops and fieldtrips. Recent data and findings "hot off the press" from laboratories, observatories and field campaigns are presented for the first time at such meetings. Hence these in-person meetings are crucially important venues which advance volcano science. But they have significant drawbacks: cost and carbon emissions. The total cost of attending an IAVCEI meeting, for example, is typically several thousand US dollars for an individual, sometimes more. Such expenses prevent many people from attending such meetings. They simply cannot afford them, nor can their organizations. Second, the carbon cost of travelling to such a meeting in terms of greenhouse gases is substantial. We propose that such information exchanges be modified in such a way to reduce cost and carbon and improve access and accessibility. A simple way to do this is to alternate in-person meetings with virtual meetings. For example, during one year the European Geophysical Union (EGU) annual meeting is in-person while the Fall American Geophysical Union (AGU) annual meeting is virtual. The next year they reverse. IAVCEI meetings could be modified in a similar fashion. Another possibility is a meeting which is partly in-person and partly online, i.e., hybrid, such as the Fall 2021 AGU annual meeting and the 2022 Cities on Volcanoes meeting in Crete. The COVID-19 pandemic is showing us how to conduct such meetings, and we strongly recommend that we not return to a pre-pandemic "business as usual" mindset for such events. At the same time, we recognize the crucially important interpersonal and mental health aspects of in-person meetings which virtual meetings do not capture.

#### Social media streams

The community continues to discover novel ways to use social media (Williams and Krippner 2018; Lowenstern et al. 2022). Many opportunities exist in the "learning" framework that we have outlined above for social media, including volcanic activity, education, EDI and meetings. Social media could be incorporated or embedded into an encyclopedia, courses and videos and films, to illustrate several examples. Social media can supply real-time information in terms of current activity at a volcano (Yute et al. 2021). For example, a course on lava dome activity could be linked to social media which is recording unrest at actively growing lava domes such as observed at Soufrière St. Vincent during December 2020–April 2021. Social media reports can be corrected and updated easily unlike a journal or book publication, although such reports are generally not peer-reviewed and can be unreliable.

### Research and using

#### Community approaches

In the past several decades, the science of volcanology has become a highly integrated and collaborative discipline. This reality is reflected in a number of initiatives including IAVCEI commissions and networks, best practices, collaborative resources and large consortia. The *IAVCEI commissions and networks* (https://www.iavceivolcano.org/commissions-networks/) provide a means for researchers to collaborate, exchange data and ideas and meet in the field and at conferences and workshops. There is commonly synergy between two or more commissions with overlapping interests. Examples of *best practices* include volcano observatory consortia for eruption forecasting, hazard communication and long-term hazard assessment (Pallister et al. 2019), risk assessment in volcanology including hazard, exposure and vulnerability (Bonadonna et al. 2018), numerical model comparison of volcanic eruptive columns (Costa et al. 2016) and aeolian remobilization of volcanic ash (Jarvis et al. 2020), among others. *Collaborative resources* are numerous today. One of the best is Vhub (https://vhub.org/), a clearinghouse which offers a wide range of materials including simulation and modeling tools, collections of data and educational resources. Many observatories maintain data (see for example http://wwwobs.univ-bpclermont.fr/SO/televolc/dynvolc/), as do the online resources of most journals. *Large consortia* address large-scale regional issues and aspects of volcanology, commonly involving researchers from a number of countries. Examples include EUROVOLC (https://eurovolc.eu/), FUTUREVOLC https://futurevolc.hi.is/) and CONVERSE (https://volcanoresponse.org/).

#### Volcano catalogues

A number of catalogues, both past and current, have been produced. These provide basic and essential information for many volcanoes on Earth. Starting in 1951, IAVCEI produced a series of catalogues by region entitled, "Catalogue of the active volcanoes of the world, including solfatara fields." The catalogue remains useful today, with many useful facts on eruption histories, eruptive styles, petrology and so forth. The Smithsonian Institution maintains a web-based compendium of data on many volcanoes (https://volcano.si.edu/), with an associated print compilation of essential information (Siebert et al. 2011). Web-based catalogues are also being actively developed and maintained, e.g., the European Catalogue of Volcanoes (https://volcanos.eurovolc.eu/#). The Springer series, Active Volcanoes of the World, includes a number of books each of which provide a focus on important individual volcanoes or groups of volcanoes (https://www.springer.com/series/10081/books). Another Springer series, Advances in Volcanology, comprises books with both a thematic and geographic focus (https://www.springer.com/series/11157/books). These various collections all focus upon subaerial volcanoes and volcanism, with a notable gap in submarine volcanoes. Filling this gap is a challenge and should be a future priority.

#### Digital elevation models (DEM's)

DEM's are a basic and fundamental element for monitoring and understanding a volcano's behavior. Without a current DEM, modern measurements and monitoring cannot be made. DEM's are fundamentally important for geophysical measurements and monitoring (Chirico et al. 2009), and they are also invaluable for physical volcanology (Albino et al. 2015), as landforms and landscapes change during eruptive activity. Modern DEM's commonly have extremely high resolution, typically one meter or even better. When a volcano is active, DEM's can be revised and updated to reflect surface changes. In turn, such updated “real-time” DEM's can be used quantitatively to reveal subsurface processes such as magma movement. In cases of relatively high DEM uncertainty, options are available, such as the stochastic approach of Favalli et al. (2005). A challenge for future DEM's is increased spatial and temporal resolution at the scale of tens of centimeters or even centimeters (Azzaro et al. 2012). A current source of DEM data can be obtained from the Shuttle Radar Topography Mission (SRTM) (https://www2.jpl.nasa.gov/srtm/).

#### Hazard and risk maps

Maps that illustrate hazard, exposure, vulnerability and risk are fundamentally important for lives, livelihoods and infrastructure (Blong 1984; Lowenstern et al. 2022). By integrating these different type of maps, an understanding of risk to primary and secondary hazards emerges for people and the environment, including both natural and developed landscapes. There is a need to assemble, in a repository and centralized fashion, maps of infrastructure, commercial and industrial property, agriculture and wildlife, along with human population distributions. A central repository of such maps produced for active and potentially active volcanoes would therefore be a remarkable resource for many stakeholders including urban planners, developers, civil defense officials, politicians and the public. Without such information, proper planning and preparation for volcanic unrest and eruptions cannot be fully accomplished. It is a significant challenge to assemble and organize a comprehensive repository of such maps, including revised and updated versions. A repository for hazard maps can be found at https://volcanichazardmaps.org/. A useful analogy is the need to assemble a repository of material safety data sheets (MSDS) in a centralized fashion for a chemistry lab with 500 chemicals, due to the threat that the chemicals and waste products may pose to humans and the broader environment.

#### Links to individual volcano repositories

The best studied, most monitored and most active volcanoes typically have extensive data resources and data collections which are available electronically. Such repositories are discussed in greater detail below; here we underline the need for links to such volcanoes. The volcanoes could be chosen on the basis of their unrest, e.g., those with lava dome growth, those with significant lahar activity, etc. A challenge is to identify the key volcanoes whose data lend themselves to this type of comparative approach. Another challenge is accessing data, which requires an open, collaborative approach and philosophy.

The World Organization of Volcano Observatories (WOVO) is a group of institutions that monitor active volcanoes. These institutions and personnel are on the front lines of assessing volcanic unrest. Currently the organizations are listed on a website (https://wovo.iavceivolcano.org/). Although somewhat dated currently, this website has important links to other information sources such as WOVOdat (https://www.wovodat.org/). The potential for increased integration among volcano observatories is extremely high. A detailed directory of each organization could be assembled, containing key links to personnel, data and other resources. Well-linked organizations could share and exchange data easily. Easy and rapid communication channels such as Zoom and Microsoft Teams could be in place and made available as needed. Using such communication resources, expert solicitations could be efficiently and rapidly accomplished using a wide range of people when volcanic unrest occurs (Lowenstern et al. 2022). A well-linked observatory network could share hardware, software and instrumentation, while an exchange program of personnel could be established to foster collaborations and best practices (see for example https://www.ird.fr/workshop-virtuel-sur-la-reponse-aux-eruptions-volcaniques-effusives). Dedicated funding for such activities would be an important achievement. The challenges and opportunities are numerous.

## An integrated knowledge-information system for individual volcano systems

**Fig. 2:**
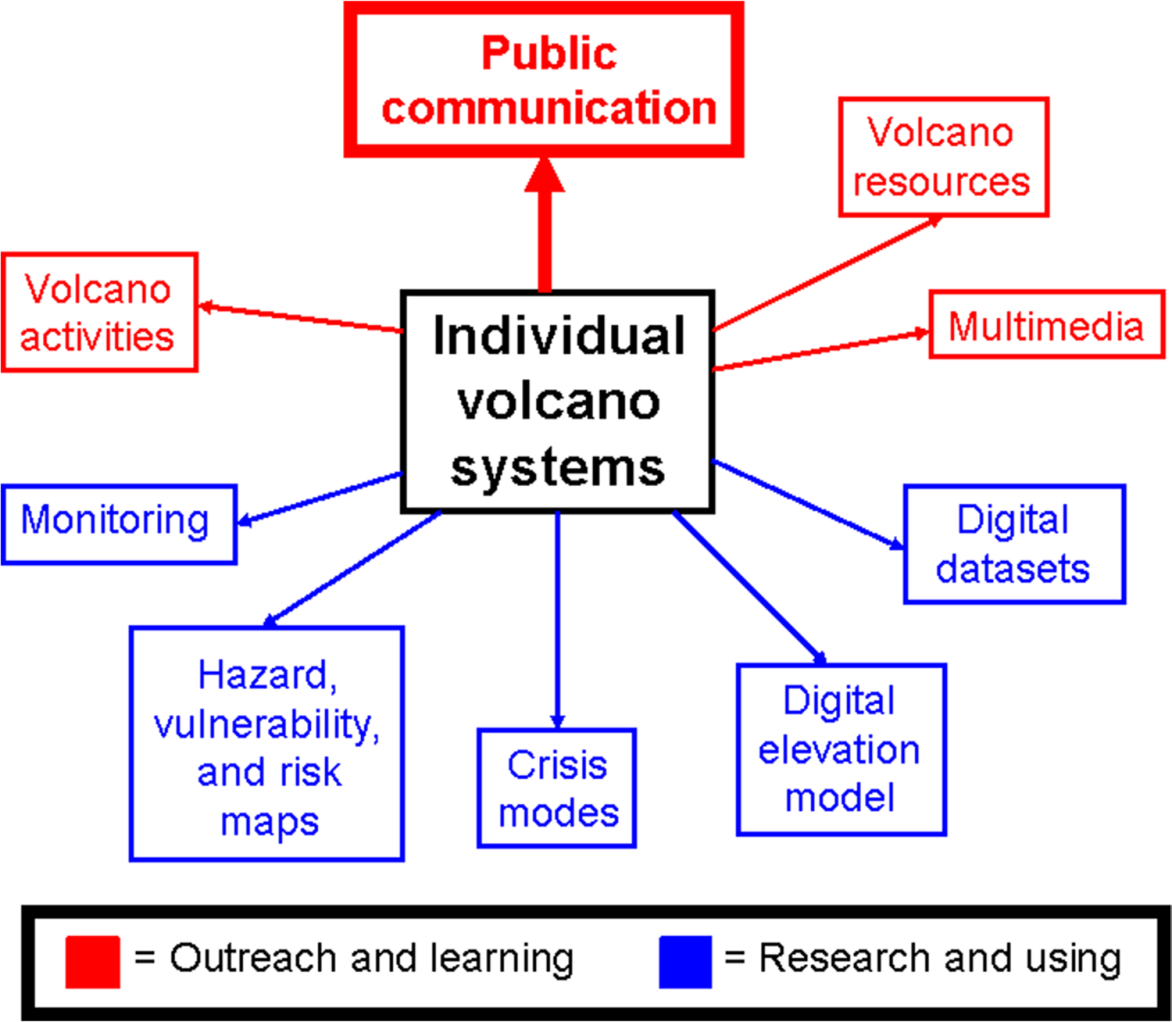
An integrated knowledge web for individual volcano systems

We now focus on individual volcano systems and propose a similar two-component structure to assembling information. The two components have clear links amongst a number of topics within these components. Although the “outreach and learning” and “research and using” components are different and distinct, they can be usefully integrated for a holistic view of a particular volcano system. The concepts are shown schematically in Fig. 2.

### Outreach and learning

#### Public communication

Providing knowledge and insight regarding a volcano's activity, its history and past unrest, and its resources is a significant challenge, requiring ongoing communication and discussion. Links to key stakeholders are an important component, as well as contact with schools and the use of social media. There is high potential for interesting feedbacks, e.g., communicating monitoring data, explaining hazard maps and the like. Helping people understand how a volcano observatory functions is also important, e.g., visiting the observatory and meeting observatory personnel, fieldtrips to monitoring sites and geoheritage sites, films and videos, etc. (see below).

#### Volcano activities

Field guides and fieldtrips are an effective means of understanding a volcano. Such guides could be written, virtual and/or multimedia in nature. Trips could include visits to observatories, museums, monitoring sites, geological features and other geoheritage sites such as hydrothermal areas, protected zones and flora and fauna. In addition, an annual public meeting on the volcano could be created, possibly with different themes from one year to the next. Such meetings could include an assessment and presentations on the "state of the volcano." In the context of these activities, tourism could be developed and promoted, in the process building good links among scientists and researchers, tourist operators and the local chamber of commerce.

#### Volcano resources

All volcanoes have interesting and varied resources, such as soils, agriculture and forests (Becerra-Ramírez et al. 2020). Many have mineral resources such as sulfur and clays. At a number of volcanoes, unusual geothermal and hydrothermal features are present. Flora and fauna are commonly abundant, and many volcanoes, in particular their upper slopes, are protected areas or reserves. These are significant resources for public communication, for visits by community and school groups and for tourism and ecotourism.

#### Multimedia

A series of films, videos and other multimedia products such as Youtube channels could be developed highlighting the different aspects of a volcano and its activity. Such a multimedia package might include the following elements: (1) focussing on the volcano's geologic history, past activity and current unrest; (2) demonstrating how scientists study and monitor the volcano, including the difficulties involved and the associated uncertainty in making forecasts; (3) documenting the volcano's resources; (4) showing how people live with the volcano, both in times of quiescence and during unrest and eruptive periods and the aftermath; (5) examining questions of EDI, illustrating how under-represented groups are involved in "non-traditional" activities such as fieldwork, monitoring and leadership. Such a package of multimedia products, especially if well structured and integrated together, could be a remarkable contribution to outreach and public understanding, and could also serve as a model for other volcanoes.

### Research and using

#### Monitoring

A number of active volcanoes are monitored using seismic, deformation, gas and webcam networks, which together provide a good view into the workings of a volcano. A very small number of volcanoes are highly instrumented; more commonly, only a few instruments monitor activity, even a single seismometer in some cases. Some active volcanoes have no instrumentation at all. Given limited resources, this is reasonable and understandable. In some observatories today, incoming data are livestreamed in real time, available to anyone. Such data streams are important for the monitoring effort and for research, also serving as outreach tools on the observatory website. This form of outreach can be supplemented with a daily briefing or discussion by a trusted delegate of the observatory.

#### Hazard maps

A repository of hazard maps for a volcano is similarly useful for monitoring, research and outreach. An archive could show various versions of past maps, with the current map or maps highlighted (Chevrel et al. 2021). Hazards could be depicted at different spatial scales, with zooms into particularly vulnerable or complex areas. Some maps are static, others are interactive. A website currently exists which incorporates many of these concepts (https://volcanichazardmaps.org/). Such a map repository could be expanded to include maps of exposure, vulnerability and risk.

#### Crisis modes

An interesting aspect to consider is to establish a digital "corner" with information and data that pertain to times when a volcano is particularly active or in crisis mode. Such a corner, which could be an online forum open in digital space, could focus on issues such as expert elicitation, conveying and understanding uncertainty to scientists, officials and the public, key monitoring data, evolving hazard maps, public communication and forecasting efforts. Various scenarios which the volcano might follow in the near to medium term future could be explored, explained and tested. This corner could remain in "sleeping" mode during normal conditions and "re-activate" as the volcano re-activates. Many observatories use this practice on a regular basis where they provide eruption updates on their websites.

#### Volcano digital elevation models (DEM's)

An up-to-date DEM of the volcano can reside digitally for all to use. The DEM would be periodically updated and revised with new imagery including drone data (De Beni et al. 2019). It could be interactive, easily usable and downloadable. It would provide a common platform for anybody working on the volcano, including researchers, officials, planners and the public. Schools could find interesting uses of the DEM for their own purposes, and in doing so, teach students the fundamentals of modern digital cartography.

#### Digital datasets

A series of datasets, including but not limited to monitoring data, meteorology, geological history, physical volcanology, petrology-geochemistry, geophysics and historical literature, are an invaluable tool for many different studies, including retrospective analysis, better understanding of how the volcano works and outreach and teaching purposes, both for scientists and the public. Where appropriate, these can be made open and accessible.

## Concluding remarks

Volcano geoheritage has greatly evolved since a time when the only sources of information about volcanoes were comprehensive books written after years of observation and research. Access to this information was limited to those with research libraries or personal collections. Many of those at risk during volcanic eruptions had few of these resources, and observations were limited to scientists from the industrial nations.

Now the world has opened up for the many resources available to those with online access—resources that include training, volcano data, advice and online observations of eruptions in real time. There are still books and they are used, but they are not limited to print runs and libraries. The new access to resources is potentially available to everyone, regardless of nationality or background. This engenders an optimistic view of the potential for volcano geoheritage.

We believe that exciting opportunities exist today to develop a new vision of volcano geoheritage for tomorrow. Clearly there are challenges about how to best implement the ideas outlined in this paper. We can imagine a number of different approaches which could help develop and jumpstart these concepts:

- An IAVCEI working group could map out a five-year implementation plan. The working group would have EDI principles embedded, both in its composition and its mapping approach. The IAVCEI Commission on Volcano Geoheritage and protected Volcanic Landscapes could be an appropriate vehicle.

- IAVCEI could provide seed funding for an initial mapping effort, in order to establish directions, priorities and mechanisms of implementation.

- As these concepts include and impact many sectors of society in different and diverse ways, donors could be approached to support the project financially.

- A number of national and regional funding agencies now have programs which specifically target outreach and education. A coordinated proposal-writing effort could be instituted among researchers from different countries.

- Some of the individual components discussed above could have their own funding mechanisms, e.g., a regional or global effort to develop and expand volcano DEM’s.

- International agencies such as UNESCO could be approached for support. Not only would the project benefit volcanology, it could also be seen as a template and have appeal for other organizations and other disciplines within the IUGG umbrella and beyond.

While our ideas might seem daunting and difficult to achieve in terms of a fully holistic approach and a full integration of the concepts presented herein, they need not necessarily be so. They could be instituted step-wise, at different scales, and progressively. They could build from small-scale to large-scale. Some of the growth could be organic, in the sense of rapid growth and acceptance after the initial stages. In conclusion, it will be interesting and exciting to observe the development of volcano geoheritage over the next decade and beyond.
